# Traumatic Lumbosacral Dislocation: Current Concepts in Diagnosis and Management

**DOI:** 10.1155/2018/6578097

**Published:** 2018-10-28

**Authors:** Andrew S. Moon, Kivanc Atesok, Thomas E. Niemeier, Sakthivel R. Manoharan, Jason L. Pittman, Steven M. Theiss

**Affiliations:** UAB Faculty Office Tower #960, 510 20th Street South, Birmingham, AL 35233, USA

## Abstract

Traumatic lumbosacral dislocation is a rare, high-energy mechanism injury characterized by displacement of the fifth lumbar vertebra in relation to the sacrum. Due to the violent trauma typically associated with this lesion, there are often severe, coexisting injuries. High-quality radiographic studies, in addition to appropriate utilization of CT scan and MRI, are essential for proper evaluation and diagnosis. Although reports in the literature include nonoperative and operative management, most authors advocate for surgical treatment with open reduction and decompression with instrumentation and fusion. Despite advances in early diagnosis and management, this injury type is associated with significant morbidity and mortality, and long-term patient outcomes remain unclear.

## 1. Introduction

Traumatic lumbosacral dislocation is a rare clinical entity, characterized by unilateral or bilateral facet dislocations causing displacement at the level of the fifth lumbar vertebra in relation to the sacrum [1]. This injury pattern is caused by high-energy mechanisms such as motor vehicle collisions, falls from height, and crush injuries and is frequently associated with severe concomitant injuries [2].

There is some discrepancy in the literature with regard to the terminology describing injuries in this region; traumatic L5-S1 spondylolisthesis [3–7], lumbosacral/lumbopelvic dissociation [8–12], suicide jumper's fracture [13], spinopelvic dissociation [14–17], and spondylopelvic dissociation [18–20] have all been used to describe a spectrum of similar injuries. The terms spinopelvic and spondylopelvic dissociation are generally reserved for a more severe injury pattern with U-type, H-type, II-type, Y-type, or lambda type sacral fractures in conjunction with bilateral sacral fracture dislocations [17]. In this injury pattern, the spine and upper sacrum displace into the pelvis, separating from the remainder of the intact pelvic ring. However, to be a true lumbosacral dislocation, there must be dislocation of the facet joints between the fifth lumbar vertebrae and the sacrum.

Sacral fractures and lumbosacral dislocations are estimated to account for 1% of spinal fractures [21]. Current literature on lumbosacral dislocations is sparse, mainly consisting of case reports and small case series. The aim of this present study was to review the current literature on lumbosacral dislocation with regard to the relevant anatomy, biomechanics of injury, classification schemes, clinical evaluation, management, and prognosis.

## 2. Anatomy and Biomechanics of Injury

The lumbosacral junction consists of the L5 and S1 vertebra, as well as the corresponding intervertebral discs and apophyseal joints. It is a well-supported region stabilized by the local paraspinous musculature and iliolumbar ligamentous complex, which connects the transverse processes of L5 to the posterior iliac wing and crest. The ligamentous structures contributing to iliolumbosacral stability include the supraspinous ligament, ligamentum flavum, interspinous ligament, iliolumbar ligament, lateral lumbosacral ligament, and the facet joint capsule. The lumbosacral joint has an increased inclination in the sagittal plane and the facets at this junction have a more vertical, frontal plane orientation [22], resisting anterior translation and making dislocation a rare injury. Several authors have suggested that preexisting spondylolysis at the level of L5 may be a predisposing factor for the disruption of the lumbosacral junction with additional trauma [23–27].

This injury pattern is considered an unstable injury with disruption of virtually all stabilizing structures in that area, and may be suspected in cases where impact occurs cranial to or directly at the level of L5-S1. The direction of dislocation may vary depending on the traumatic force vector, and includes anterior, anterolateral, lateral, and posterior dislocations. Anterior dislocations, resulting in L5 anterior to S1, are most common [3, 23], while posterior dislocations are typically associated with more severe neurological injuries [3, 28–31].

Watson-Jones first described this injury pattern in 1940, and suggested forced hyperextension as the mechanism of injury [32]. However, Roaf et al. in 1960 demonstrated experimentally that the forces responsible for anterior dislocation were a combination of hyperflexion, axial rotation, and compression [33]. To date, the literature has shown hyperflexion to be the most common mechanism of injury [23]. However, isolated hyperflexion is unlikely to produce this type of injury in the lumbar spine [33]. Other contributing forces may include compression [6, 7, 34, 35], rotation [36–42], distraction [43, 44], translation [45–47], lateral translation [48–50], lateral bending [25, 36, 51], and direct traumatic vectors [44, 52].

Each variant of a lumbosacral dislocation is thought to occur through a slightly different combination of forces [7, 25, 28–32, 49, 53–56], and while multiple mechanisms have been postulated, no biomechanical study directly supporting a given mechanism of injury has been performed.

## 3. Classification

**Figure 1:**
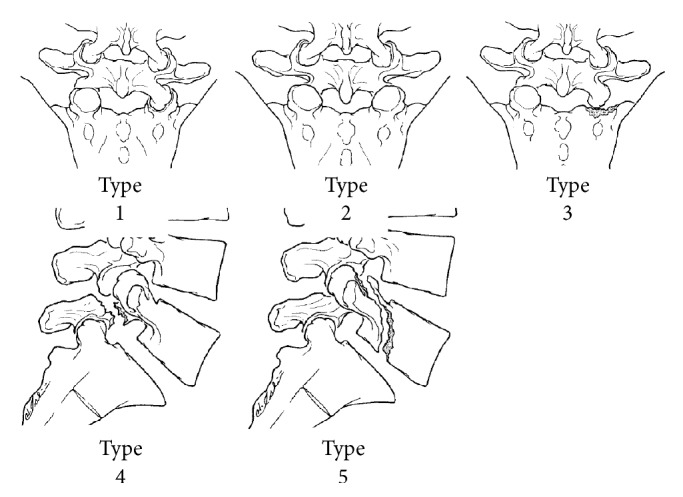
Classification of fracture-dislocation of the fifth lumbar vertebra according to Aihara et al. [1].

In 1998, Aihara et al. proposed a classification scheme specifically for fracture dislocations of the fifth lumbar vertebra based on the existing literature (Figure 1) [1]. Type 1 involved unilateral lumbosacral facet dislocation with or without facet fracture, with an intact contralateral facet. Type 2 involved bilateral lumbosacral facet dislocation with or without facet fracture. Type 3 involved unilateral lumbosacral facet dislocation and contralateral lumbosacral facet fracture. Type 4 involved dislocation of the body of L5 with bilateral fracture of the pars interarticularis (acute spondylolytic spondylolisthesis). Type 5 involved dislocation of the body of L5 with fracture of the body and/or pedicle with or without injury of the lamina and/or facet. This first attempt at a classification scheme did not distinguish between intact and unilateral/bilateral fractured facets, and other classification schemes based on varying anatomic factors exist in the literature [57, 58].

## 4. Clinical Evaluation

Given the substantial, high-energy trauma necessary for this injury, there are typically a variety of associated injuries involving bony, ligamentous, soft tissue and/or neurovascular elements [21], and the diagnosis may be easily overlooked on initial evaluation. Shen et al. reported that 10% of reported lumbosacral fracture dislocations were not initially recognized, though these were in patients where X-ray was the primary imaging modality [36]. Clinical presentation varies widely, [59–78] and may include severe lower back pain with exam findings such as flank hematomas, abrasions or palpable step-offs of the spinous processes.

Associated injuries are likely to occur locally, but may also involve other body cavities such as the abdomen, pelvis, thorax, and cranial cavity [21, 79]. Bony injuries may include vertebral fractures of the transverse processes, spinous processes, and sacral promontory, as well as distant fractures such as in the ribcage or femur [36]. Local soft tissue involvement includes the supraspinous ligaments, paraspinous musculature, facet joint capsules, dura and intervertebral disc [80, 81].

Typically associated neurological injuries include cauda equina syndrome and disruption of the lumbosacral plexus [36, 46, 82, 83]. Neurologic findings on exam may include hypoesthesia of the lower extremities, radiculopathy, bowel dysfunction, and urinary retention [84]. S1 is the most frequently affected nerve root [22, 39, 44, 49, 85], and more serious neurological injuries include paraplegia, although this is rare [81]. Neurologic compromise, as well as persistent neurologic deficits postoperatively, is more likely in bilateral dislocations or dislocations with fractures [25, 35, 58, 71, 72, 86].

There is a wide range of reported rates of neurological injury in the literature. Aihara et al. reported a 68.4% rate of neurologic deficit in 57 cases [1], while only 3 out of 11 patients (27.3%) in the series by Vialle et al. demonstrated neurological injury [58]. Grivas et al. reported a 58% rate of neurologic deficit for all lumbosacral fracture dislocations [2], while Arandi et al. found an 89% rate of neurological injury for complete lumbosacral dislocations [59].

## 5. Imaging

Initial work-up with high-quality standard radiographic studies will demonstrate an abnormal relationship between the lumbosacral facets. Clues to this pathology on the anteroposterior view include transverse process fractures (sentinel fractures), obliquity of L5 on sacrum, widening of the paravertebral soft tissue lines, widening of the interpedicular distance, and rotational deformity of the spinous processes [22, 27, 36, 85, 87]. On the lateral view, there may be an increased interspinous distance, kyphosis of L5 on S1, anterior or posterior subluxation of L5 on S1, anterior narrowing of height of disc space, disrupted spinolaminar lines, or amplification of lumbar lordosis [22, 27, 36, 87].

**Figure 2:**
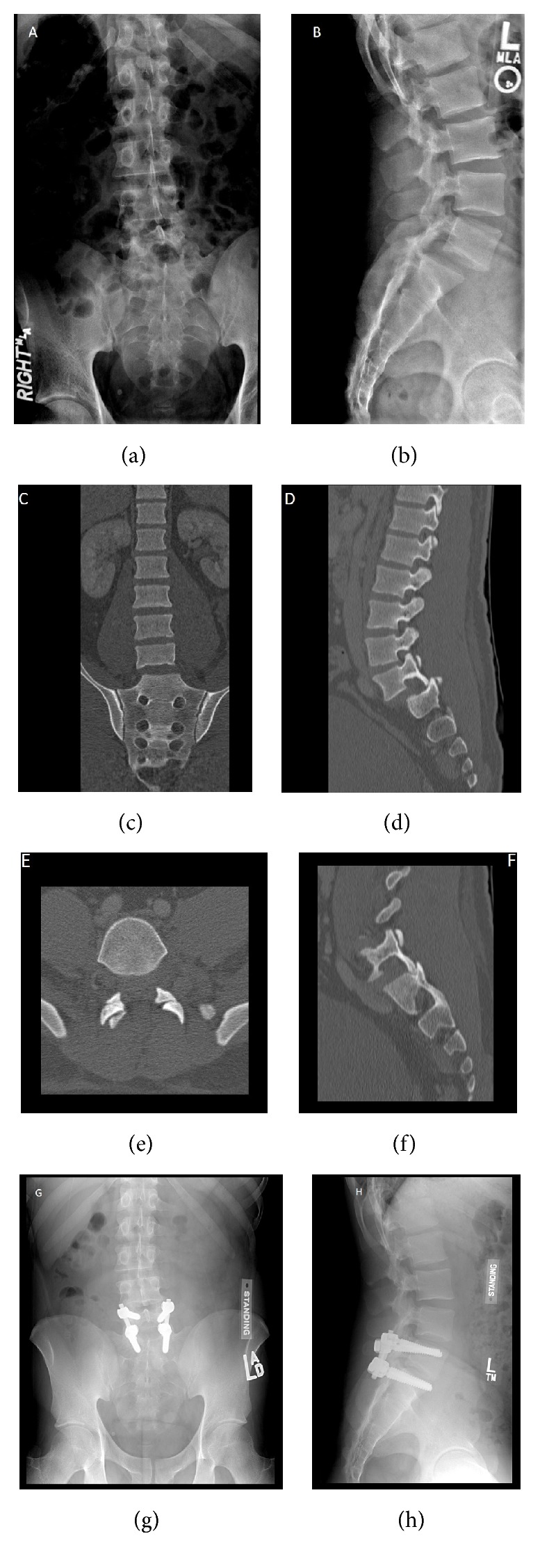
Imaging of a 25-year-old male patient who was involved in an all-terrain vehicle accident. He was ejected from the vehicle and presented with low back pain and intermittent bilateral lower extremity radicular pain with paresthesia. Figures (a) and (b) demonstrate anteroposterior and lateral radiographs, respectively. Coronal CT shows minimal lateralization of L5 over S1 (c). Sagittal view shows anterior dislocation of L5 over S1 with jumped facets (d). Axial (e) image cut through the same level as the sagittal image (f) shows bilateral jumped facets at L5-S1. The patient underwent posterior spinal instrumentation and fusion of the L5 and S1 vertebrae using pedicle screws and rods. Postoperative anteroposterior (g) and lateral (h) images demonstrate a reduced L5-S1 joint. (Courtesy of University of Alabama at Birmingham, Department of Orthopaedic Surgery, Spine Fellowship Program, Birmingham, Alabama, USA).

Advanced imaging modalities are now routinely used in virtually all high-energy trauma patients and will readily demonstrate the injury (Figure 2). A computed tomography (CT) study allows for visualization of injuries to the posterior elements and locked or fractured facet dislocations with displacement of L5 on S1 [22, 62, 88]. CT may show associated fractures, such as laminar or sacral fractures, as well as a “naked facet sign” on the axial plane, due to the L5 facets passing superiorly over the facets of S1 [22, 63, 87, 89, 90]. This gives the CT scan an image of empty or perched facets, and is indicative of facet dislocation. A magnetic resonance imaging (MRI) study will also demonstrate the dislocation, along with other local injuries including disc herniation, dural tears, torn discs, root compression, and degree of musculoligamentous injury. MRI can be instrumental in localizing sites of neural compression [22, 62, 87].

## 6. Management

Initial management includes appropriate evaluation, stabilization and resuscitation measures according to standard Advanced Trauma Life Support (ATLS) protocol, and emergent injuries should be treated first in order of priority [91]. There are a few published experiences with nonoperative management with techniques including closed reduction, traction and immobilization [26, 49, 56, 77, 92–95]. To the authors' knowledge, the last case report of an adult treated conservatively was published in 2000, with the authors opting for conservative treatment due to the patient's delayed presentation of three months [92].

In the pediatric population, nonsurgical treatment remains a consideration with any spinal condition due to concerns of disproportional growth of the anterior spine after isolated posterior fusion resulting in a progressive, iatrogenic deformity frequently referred to as the “crank-shaft phenomenon.” [96] However, with closed reduction and immobilization, studies have documented risk of secondary neurological injury during external reduction maneuvers [46, 56, 58, 93]. In addition, prior reports have documented an increased risk of progressive back pain, deformity, and neurologic deterioration with conservative treatment [4, 26, 31].

Acute lumbosacral dislocations are unstable, and a growing body of literature recommends early surgical reduction with instrumentation [1, 59, 71, 81, 92, 97, 98]. Historic techniques for instrumentation have included a wide range of constructs including interspinous screws, posterior articular screws, sublaminar wiring, Harrington hooks and rods, and osteosynthesis with posterior plates or with Cotrel-Dubousset-type instrumentation [23, 25, 27, 30, 40, 54, 64, 84, 99–104].

Currently, treatment should consist of pedicle screws in L5 and S1, assuming the pedicles at these levels are intact. While this short segment construct may be sufficient in patients with good facet apposition following reduction, fixation may need to be extended proximally to L4 or distally to the pelvis when bony support is poor after reduction. The lumbosacral canal should be examined intraoperatively for any bone or disc fragments, if MRI indicates neurocompression [3, 54, 58]. Spinal cord monitoring can be used to confirm intact peripheral nerve function during reduction maneuvers and significant distraction should be avoided during reduction.

All dislocation injuries should also be treated with fusion. While there is little literature describing the superiority of one fusion method over another, options include posterior arthrodesis [1, 59], circumferential arthrodesis [27, 70, 72], and interbody fusion, which is often used in cases of significant disc disruption [24, 64, 99]. Partial facetectomy may be performed in patients with traumatic lumbosacral dislocation to facilitate reduction [34, 46, 70, 85, 99], although intact apophyseal joints are preferred to prevent redislocation [34].

Numerous case reports support decompression in patients with evidence of neurologic compromise [1, 59, 71, 81, 92, 97, 98]. The authors of the current study suggest surgical decompression is patient-dependent, and recommend selective decompression based on the patient's clinical exam and sites of neurologic compression as evidenced on MRI. In cases of cauda equina syndrome or delayed reduction, decompressive laminectomy may be performed [40, 43, 70, 83]. However, this may lead to increased instability and is not indicated in the absence of neurologic compromise [68, 77, 85].

## 7. Prognosis and Complications

Although there have been a few reports of satisfactory outcomes after nonoperative management [56, 92, 93], many patients initially treated conservatively eventually required fusion due to progression of listhesis and/or neurological deficit [5–7, 22, 40, 51, 54]. However, even with surgical intervention there may be residual disability and permanent neurological dysfunction [1, 98, 100–103]. The degree of residual translational displacement and kyphosis postoperatively may be associated with clinical outcomes following surgery. Perioperative surgical complications include infection and wound dehiscence, not unlike other surgeries in this region. Additional complications include mechanical issues such as instrumentation failure that can occur late, requiring reoperation years after the initial surgery [68, 92, 102]. Adelved et al. published long-term results in a small series of patients with traumatic lumbosacral dissociation, showing that functional impairments, pain, and poor patient-reported health were common, along with high rates of neurologic, urinary, and sexual dysfunction [8]. Conversely, De lure et al. demonstrated successful long-term clinical outcomes in a small cohort of patients who underwent lumboiliac fixation for lumbosacral dislocation injuries [92]. Long-term prognosis is unclear due to the small number of reported cases with limited follow-up and heterogeneous results.

## 8. Summary

Traumatic lumbosacral dislocation is a rare injury pattern resulting from high-energy trauma. It often presents with multiple concomitant injuries, and may be easily overlooked on initial evaluation. Acute complete dislocations are highly unstable, three-column injury patterns, requiring surgical intervention with open reduction and internal fixation. Early diagnosis and treatment are likely to improve clinical outcomes. Despite advances in diagnosis and management, these injuries are associated with significant morbidity and mortality.
